# Deciphering the role of reactive oxygen species in idiopathic asthenozoospermia

**DOI:** 10.3389/fendo.2025.1505213

**Published:** 2025-05-21

**Authors:** Zilong Wang, Dandan Li, Guoyi Zhou, Zhen Xu, Xinkun Wang, Senbao Tan, Zhenghao Li, Xiaoli Li, Changze Song, Song Yuan

**Affiliations:** ^1^ Department of Andrology, The Seventh Affiliated Hospital, Sun Yat-sen University, Shenzhen, China; ^2^ Department of Burns and Plastic Surgery, The Seventh Affiliated Hospital, Sun Yat-sen University, Shenzhen, China; ^3^ Department of Radiation Therapy, Dongguan Hospital of Guangzhou University of Chinese Medicine (Dongguan Traditional Chinese Medicine Hospital), Dongguan, China; ^4^ Surgery and Anesthesia Center, The Seventh Affiliated Hospital, Sun Yat-sen University, Shenzhen, China

**Keywords:** reactive oxygen species, idiopathic asthenozoospermia, oxidative stress, sperm motility, antioxidants, male infertility

## Abstract

Asthenozoospermia is a severe condition characterized by abnormal sperm motility, contributing to 50% of male infertility cases. Idiopathic asthenozoospermia refers to a form of this condition with no identifiable causes through routine clinical examinations, potentially linked to apoptosis and oxidative stress induced by excessive reactive oxygen species (ROS). At low concentrations, ROS positively influence physiological processes, including sperm mature and motility. However, elevated ROS levels can harm human spermatozoa through oxidative stress, primarily due to the absence of effective DNA damage repair mechanisms and inadequate antioxidant defenses. In this review, we summarize the physiological and pathophysiological roles, endogenous and exogenous sources, and therapeutic strategies related to ROS in idiopathic asthenozoospermia. Ultimately, maintaining a proper balance between ROS concentrations and antioxidants is crucial for ensuring male reproductive health.

## 1 Introduction

Sperm motility is a crucial capability of human spermatozoa necessary for their journey across the female genital tract post-ejaculation (1), with progressive motility (PR) serving as a key metric (2, 3). In recent years, sperm parameters have witnessed a declining trend, especially with a sharp drop in sperm motility, which, in severe cases, leading to male infertility in severe instances (4). Currently, male infertility is responsible for the 14% of couples experiencing fertility issues (1, 2). Asthenozoospermia (AZS) is a severe condition characterized by abnormal sperm motility, defined by progressive motility of less than 32% (PR<32%) among sperm parameters (2, 3). The majority of patients with male infertility also present with asthenozoospermia. The common causes of AZS include varicocele, endocrine abnormalities, environmental factors, inflammation, drug-induced injury, and certain underlying diseases (5, 6). Nevertheless, in numerous cases, routine clinical examinations fail to identify clear causes, leading to a classification of idiopathic AZS (iAZS) (5).

The exact pathogenesis of iAZS remains unclear, but it is currently believed that excessive reactive oxygen species (ROS) leading to apoptosis and oxidative stress is a key factor in its development. ROS is a group of highly reactive oxygen-containing molecules that include superoxide anion (O_2_^-^), hydrogen peroxide (H_2_O_2_), hydroxyl radicals (OH^-^), and singlet oxygen (^1^O_2_) (7). Due to their short half-life (8), they cannot be directly detected in human specimens. The OH^-^ is particularly unstable and rapidly reacts with nearby biomolecules. Furthermore, H_2_O_2_ is a predominant form of ROS capable of crossing cell membranes to exert effects beyond cellular boundaries (9).

The intracellular levels of ROS are closely regulated by various synthesis and degradation pathways. Maintaining physiological levels of ROS is critical for redox regulation involved in processes such as repair, survival, and differentiation (10). However, when ROS are produced in excess, they can damage sperm cells, leading to impaired motility, DNA fragmentation, and cellular apoptosis, significantly affecting male fertility (11). Additionally, excessive ROS can induce lipid peroxidation in the sperm plasma membrane, which is rich in polyunsaturated fatty acids, disrupting membrane integrity and impairing sperm function and morphology (12). While ROS are often considered detrimental, they also play a vital physiological role in sperm function (13, 14). In spermatozoa, these molecules play essential roles in sperm capacitation, acrosome reaction, and fertilization (15). The challenge lies in the delicate balance between the beneficial and harmful effects of ROS (16). The role of ROS in idiopathic asthenozoospermia remains unclear (17). Furthermore, the mechanisms for maintaining the dynamic balance of ROS in sperm to manage oxidative stress in idiopathic asthenozoospermia require further investigation.

Idiopathic asthenozoospermia (iAZS) may be linked to apoptosis and oxidative stress caused by excessive ROS (18, 19). Nevertheless, the exact pathogenesis of iAZS remains unclear. This review explores the sources of ROS, their physiological and pathological roles in sperm motility, and potential therapeutic strategies targeting ROS in iAZS. By investigating these aspects, we offer new insights for the clinical management of iAZS and provide a comprehensive framework for understanding the complex interplay between ROS and sperm function.

## 2 Physiological roles of low concentrations of ROS in sperm maturation and motility

**Figure 1:**
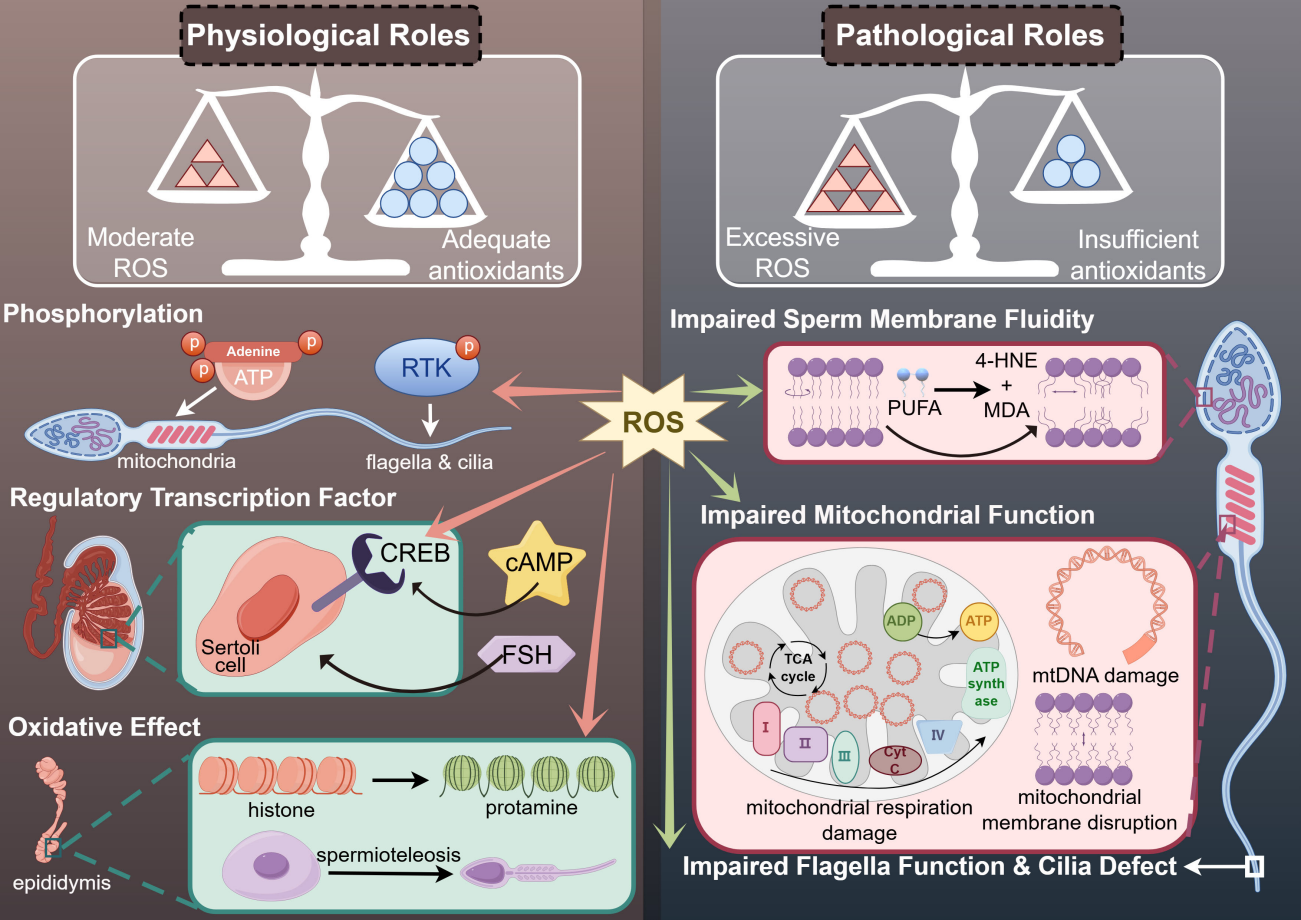
The physiological and pathological roles of ROS in human sperm motility.

At low concentrations, ROS positively influence physiological processes such as spermatogenesis, sperm motility, and fertilization (20, 21). This process might be associated with phosphorylation, the expression of regulatory transcription factor and oxidative effects (**Figure 1**) (22).

### 2.1 Phosphorylation

During spermatogenesis, the DNA replication process of meiosis relies on energy supplied through oxygen consumption and ROS generation in mitochondria, which also provide the necessary ATP for sperm motility (23). The proliferation of spermatogonia and the differentiation of spermatocytes into spermatozoa, including the formation of sperm flagella crucial for motility, depend on various internal environmental factors (24). These factors include receptor-tyrosine kinase (RTK) phosphorylation signaling pathways, which are mediated by ROS in some somatic cells (25). Therefore, physiological concentrations of ROS play beneficial roles by modulating phosphatases and facilitating phosphorylation (**Figure 1**).

### 2.2 Regulatory transcription factor

ROS at appropriate concentrations play a crucial role in the transcriptional processes of spermatogenesis by functioning as regulatory transcription factors (26). Several sex hormones, including follicle-stimulating hormone (FSH) and luteinizing hormone (LH), are also involved in the mechanism of transcriptional regulation (27). In the testes, Sertoli cells are the primary targets of FSH (28). The cAMP response element-binding protein (CREB), whose receptors are activated by optimal levels of ROS, serves as a pivotal transcription factor within Sertoli cells under the influence of FSH-mediated signaling pathways (29). However, the role of ROS as regulatory transcription factors varies distinctly from that of various sex hormones (**Figure 1**) (30).

### 2.3 Oxidative effect

Sperm maturation in the epididymis is influenced by the oxidative effects of ROS (22). Due to the lack of histone packaging, spermatozoa struggle to maintain the integrity of their genetic material through DNA damage repair processes (21). Consequently, the histone-to-protamine replacement during spermiogenesis provides an alternative mechanism for maintaining genetic stability, which results from the oxidation of small nuclear thiol group proteins in protamine (31). Additionally, ROS, as oxidants derived from redox reactions, are implicated in the formation of chromatin packaging and the mitochondrial capsule—a protective cover surrounding the chromatin and mitochondria (32). Improper formation of these structures can compromise both the integrity and energy generation required for sperm motility, leading to functional impairments in spermatozoa (**Figure 1**) (33, 34).

## 3 Pathophysiological roles of ROS in idiopathic asthenozoospermia

Sperm motility is an essential capability of human spermatozoa required for their journey through the female genital tract post-ejaculation (1). Due to the lack of adequate oxygen radical-scavenging enzymes in their cytoplasm, human spermatozoa are highly vulnerable to oxidative stress induced by reactive oxygen species (ROS), leading to idiopathic asthenozoospermia without a clear etiology (35). ROS produced by mitochondrial complex I can cause mitochondrial dysfunction through peroxidation in the mid-piece of spermatozoa, resulting in a rapid depletion of ATP, which adversely affects sperm motility (36, 37). The primary sites of ATP generation in human spermatozoa are the mitochondria, the central part of the flagella, and the sperm head (38). The key metabolic pathways involved are oxidative phosphorylation and glycolysis (18). ATP production via oxidative phosphorylation primarily occurs in the mitochondrial respiratory chain complexes through respiration, while glycolysis takes place in the central part of the flagella (**Figure 1**) (39, 40).

### 3.1 Impaired mitochondrial function

The specific activity of mitochondrial enzymes, which depend on the mitochondrial electron transfer chain complexes (ETCs), can influence sperm motility and potentially lead to idiopathic asthenozoospermia (41). Sperm motility has been shown to correlate with oxygen consumption and the efficiency of mitochondrial respiration. Several inhibitors of ETCs have been observed to impair sperm motility. Complex I in the mitochondria is particularly sensitive to excessive ROS (42), and this sensitivity arises because ROS generated through unsaturated fatty acids inhibit complex I (42). Meanwhile, the absence of mitochondrial protein OPA1 leads to disorganization of mitochondrial cristae structure and impaired assembly of ETC Complexes I, III, IV, and V, but does not affect the assembly of Complex II. OPA1 plays a role in the accumulation of mitochondrial ROS and lipid ROS induced by cysteine deprivation. Additionally, ferroptosis is a form of iron-dependent non-apoptotic cell death primarily triggered by the accumulation of intracellular iron and lipid peroxidation. Mitochondria-targeted antioxidants such as SkQ1 and redox mediators like methylene blue can inhibit the production of ROS in Complex I of the mitochondrial electron transport chain, preventing mitochondrial lipid peroxidation and ferroptosis (13).

The primary characteristics of non-motile sperm include the disruption of the mitochondrial dysfunction (43–45). Mitochondrial DNA (mtDNA) damage resulting from interactions between nitric oxide (NO) and superoxide (O_2_⁻) can also affect sperm motility and function (12, 46). And mtDNA repair is inadequate because of the complete absence of nucleotide-excision repair pathways (47, 48). Mitochondria are crucial for the energy metabolism of sperm, primarily generating ATP through oxidative phosphorylation (OXPHOS) to fuel sperm motility (49). Damage to mitochondrial DNA can result in OXPHOS dysfunction (50), which in turn impairs ATP production and leads to reduced sperm motility (36, 37). Additionally, mitochondrial DNA damage can lead to excessive production of reactive oxygen species (ROS), with elevated ROS levels triggering oxidative stress that further impairs sperm function (51). Such damage may also interfere with the expression and function of mitochondria-related proteins. For instance, mitochondrial transcription factor A (TFAM) is critical for regulating mitochondrial DNA replication and transcription. Abnormal TFAM expression may be linked to mitochondrial DNA damage, consequently affecting sperm vitality (52). Moreover, mitochondrial DNA damage can compromise the structural integrity of mitochondria, resulting in reduced sperm motility (53). Finally, mitochondrial DNA damage can influence sperm survival and function by affecting apoptotic pathways. Research indicates that mitochondrial dysfunction may activate apoptotic signaling pathways, leading to sperm cell death (54). Therefore, mitochondrial DNA damage impacts sperm vitality through various mechanisms, including disruptions in energy metabolism, oxidative stress, alterations in mitochondrial membrane potential, abnormal protein expression, and apoptosis. These interconnected pathways collectively result in reduced sperm vitality, ultimately affecting male fertility.

Furthermore, ROS can compromise the integrity of mitochondrial membranes, potentially activating apoptotic signaling cascades and promoting the release of cytochrome C (55, 56). Apoptosis in spermatozoa is typically initiated by oxidative stress and lipid peroxidation, leading to the production of mitochondrial ROS. This cascade results in a rapid loss of sperm motility, followed by caspase activation and the exposure of phosphatidylserine on the sperm surface (57). The Sperm Chromatin Structure Assay (SCSA) and active Caspase-3 levels correlate with the rate of motility decline post-ejaculation. Elevated levels of these markers suggest a faster decline in motility, indicating that apoptosis significantly impacts sperm vitality (58, 59). The phosphoinositide 3-kinase (PI3K) signaling pathway plays a role in regulating sperm apoptosis. Inhibition of PI3K activity triggers an apoptotic cascade characterized by loss of motility and oxidative DNA damage. Thus, impaired mitochondrial function due to mtDNA damage and mitochondrial apoptosis may be responsible for reduced sperm motility and idiopathic asthenozoospermia (**Figure 1**) (45).

### 3.2 Impaired sperm plasma membrane

Sperm plasma membrane may be the major target site of ROS through cascade signaling reaction (60). ROS affects the fluidity and integrity of sperm plasma membrane (12). The membrane fluidity of human spermatozoa depends on the polyunsaturated fatty acids (PUFA) in the sperm plasma membrane (61). Excessive ROS converts PUFA into 4-hydroxynonenal (4-HNE) and malondialdehyde (MDA), byproducts of LPO, to destroy the membrane fluidity of spermatozoa. Meanwhile, the generation of 4-HNE and MDA also impaired mitochondrial electron transfer chain complexes, which resulted in reducing ATP production and corresponding sperm motility, and further increased ROS from mitochondria as a result of oxidative stress (62). Therefore, ROS causes damage to membrane fluidity of human spermatozoa through the generation of MDA and 4-HNE, which in turn lead to idiopathic asthenozoospermia (**Figure 1**) (63). What’s more, loss of glutathione, a kind of antioxidants in the midpiece of spermatozoa, may also contributes to idiopathic asthenozoospermia (64).

In addition, lipid peroxidation (LPO) induced by excessive ROS could also adversely affect the fluidity of the sperm plasma membrane through oxidative stress (65). This process can result in the complete inactivation of membrane enzymes, subsequently leading to sperm DNA damage (57). Enzymes on the sperm membrane, such as phospholipase C (PLC) and phospholipase D (PLD), play crucial roles in regulating intracellular signal transduction and membrane lipid metabolism (66). Dysfunction of these enzymes can lead to disordered membrane lipid metabolism, increased ROS production, and consequently, oxidative stress and sperm DNA damage (67). For instance, overactivation of PLC can lead to an increase in intracellular calcium ion concentration, which in turn activates a series of downstream signaling pathways and increases ROS production (66). These ROS can attack the unsaturated fatty acids on the sperm membrane, triggering lipid peroxidation reactions that disrupt the membrane’s integrity, ultimately leading to sperm DNA damage (67). Additionally, when the functions of antioxidant enzymes such as superoxide dismutase (SOD), catalase (CAT), and glutathione peroxidase (GPX) are inhibited or their activities are reduced, ROS levels rise, leading to lipid peroxidation of the sperm membrane and consequently affecting the integrity of sperm DNA, resulting in decreased sperm motility (68).

### 3.3 Sperm DNA fragmentation

In spermatozoa, the integrity of DNA is crucial for protecting genetic material from environmental damage. Uncompacted DNA, due to its open structure, is more susceptible to attack by ROS, prompting mitochondria to produce apoptosis-inducing factor (AIF) and sperm DNA fragmentation (SDF) (69). DNA damage is assessed by the DNA frag-mentation index (DFI) rate using the comet assay, the sperm chromatin dispersion assay, terminal deoxyuridine nick end labeling (TUNEL) assay, and sperm chromatin structure assay (70, 71). Studies have shown that the sperm DFI is significantly negatively correlated with progressive sperm motility (72, 73). Specifically, for every 10% increase in DFI, the probability of male conception may decrease by up to 30% (1, 74, 75). One study found that a sperm DFI greater than 30% is a threshold for a significant decline in conception rates; when DFI exceeds 30%, the success rates of natural conception and intrauterine insemination (IUI) are nearly zero (1, 74, 75). SDF is a type of DNA damage that occurs under conditions involving sperm caspase and endonuclease activity, which subsequently affects the transition from histone to protamine (76). The reduction in sperm motility is also linked to the inhibition of this histone-to-protamine transition (77, 78). In patients with asthenozoospermia, the expression levels of protamine are typically lower, which may affect the motility and fertilization capacity of spermatozoa (79). Thus, SDF is one of the manifestations of idiopathic asthenozoospermia.

The relationship between SDF and reduced sperm motility is complex, involving various molecular mechanisms. Studies suggest that oxidative stress is a primary factor contributing to SDF. It leads to the excessive production of ROS within sperm cells, which attack DNA, causing strand breaks and thereby increasing DNA fragmentation (80). Moreover, oxidative stress can impair mitochondrial function, disrupting energy metabolism and resulting in diminished sperm motility (52). The chromatin packaging state of sperm is another critical factor. Research has shown that sperm with poorly packaged chromatin are more prone to DNA fragmentation and cell death during freeze-thaw processes (81). This inadequate chromatin packaging may be linked to insufficient protamine levels, which are essential for the high degree of chromatin compaction in spermatozoa (82). Additionally, sperm DNA fragmentation is correlated with the age of the sperm. As age advances, the integrity of sperm DNA declines, and DNA fragmentation increases. This is possibly due to age-related oxidative stress and a decline in antioxidant defense mechanisms (83).

### 3.4 Impaired flagella function

Human spermatozoa can utilize various carbohydrates to generate ATP necessary for sperm motility. However, even in the absence of impaired mitochondrial function, inhibition of glycolysis can also affect sperm motility (84). The flagella, which constitute most of the sperm tail structure, play a crucial role in facilitating sperm motility. For instance, cAMP can promote the phosphorylation of protein kinase A (PKA) in sperm flagella (85), which is followed by the activation of tyrosine kinases and the phosphorylation of tyrosine residues in sperm proteins, including AKAP3, AKAP4, FSIP2, CABYR, and VCP (86, 87). The cyclic AMP (cAMP)-mediated PKA signaling pathway in sperm has been shown to be downregulated due to oxidative stress in idiopathic asthenozoospermic males (84). Levels of cAMP are positively correlated with sperm motility (88). cAMP is activated by intracellular soluble adenylyl cyclase (sAC), which is encoded by the ADCY10 gene under the stimulation of Ca^2+^, and is essential for sperm motility (89). Mutations in the ADCY10 gene can lead to a decline in sAC, resulting in idiopathic asthenozoospermia[106].

Meanwhile, AKAP3, a structural protein acting as the regulatory subunit of PKA, forms the fibrous sheath, maintaining the structural integrity of the sperm flagella in collaboration with AKAP4 (90). A deficiency in AKAP3 may impair sperm motility due to the abnormal accumulation of DNA and RNA metabolites (91). Mutations in AKAP3 and AKAP4 can lead to structural abnormalities in the sperm tail’s flagella (91). FSIP2 anchors cAMP-mediated PKA into AKAP4 to sustain sperm motility. Mutations in FSIP2, characterized by the absence of CPC, IDA, and ODA, can cause idiopathic asthenozoospermia due to the lack of AKAP4 protein (92). Thus, idiopathic asthenozoospermia can be monitored through AKAP3, AKAP4, and FSIP2 within the cAMP/PKA signaling pathway (93).

Additionally, the glycolysis process in the central part of the flagella provides sufficient ATP for its function to support sperm movement (94). Researchers have found that GPI, MDH1, PGAM1 and PGAM2A, the glycolysis-mediated proteins, were downregulated in the spermatozoa of patients with iAZS (95). Meanwhile, a significant number of glycolytic enzymes, including lactate dehydrogenase, phosphofructokinase, hexokinase, glyceraldehyde-3-phosphate dehydrogenase (GAPD), and phosphoglucose isomerase, have been identified in the sheath of the sperm flagella, maintaining its function (38). Additionally, in seminal plasma, researchers have demonstrated that citric acid, malic acid, succinic acid, which are associated with energy metabolism, and pyruvate were collectively reduced in the iAZS group, while lactate levels were elevated (96). These findings indicate a shift towards anaerobic glycolysis, resulting in decreased production of ATP compared to aerobic catabolism via the tricarboxylic acid cycle (96). This metabolic alteration likely contributes to reduced sperm motility (**Figure 1**).

## 4 The sources of ROS in human ejaculate

ROS is produced through both endogenous and exogenous pathways and play critical roles in sperm function. Human spermatozoa are significant sites of cellular ROS production (97, 98). Meanwhile in the context of iAZS, endogenous ROS are often produced in excess.

### 4.1 Endogenous sources and their effects on sperm motility

Human ejaculate contains a diverse array of round cell types, including human spermatozoa at various developmental stages, leukocytes, and epithelial cells (97, 98). The ROS contributed by these cells constitute the majority of the endogenous ROS pool, which is predominantly found in seminal plasma. Among them, immature spermatozoa and leukocytes, such as neutrophils and macrophages, are considered major endogenous sources of ROS (99, 100). The mechanisms of endogenous ROS generation in immature spermatozoa and leukocytes lied in two primary pathways: the reduced nicotinamide adenine dinucleotide (NADH)-dependent oxidoreductase system in the mitochondria and the nicotinamide adenine dinucleotide phosphate (NADPH) oxidase system located in the spermatozoa plasma membrane (101).

The mitochondrial oxidoreductase system is responsible for the majority of ROS production within human spermatozoa, primarily due to the abundance of mitochondria, which supply continuous energy for sperm motility (101). Mitochondrial ROS generation is fundamentally linked to the process of respiration. NADH oxidoreductase plays a critical role by catalyzing the oxidation of O_2_ to O_2_^-^, a precursor of sperm ROS, while transferring electrons from NADH to coenzyme Q10 (CoQ10) within the mitochondrial respiratory chain (102). If mitochondrial O_2_ concentrations are elevated, coupled with increased respiratory rates, more superoxide is released (103).

In the plasma membrane of human spermatozoa, NADPH oxidase also contributes to the transformation of O_2_ to superoxide (104). NOX5, a type of NADPH oxidase located in the acrosome and midpiece region of human spermatozoa, is activated through the binding of Ca^2+^ to its N-terminal cytoplasmic domain (105). These conformational changes facilitate the generation of superoxide, making the ROS generated by NOX5 as a major component of reactive oxygen species in human spermatozoa (105).

#### 4.1.1 Immature spermatozoa

The synthesis of ROS in semen is influenced by the maturation level of spermatozoa (106). During their development and maturation, damaged or immature spermatozoa may retain residual cytoplasmic droplets, which are remnants of spermatogenesis. These droplets contain glucose-6-phosphate dehydrogenase (G6PD), a cytosolic enzyme that produces an excess of NADPH. This NADPH acts as a substrate for NADPH oxidase, facilitating the conversion of O_2_ to O_2_^-^ (107).

**Figure 2:**
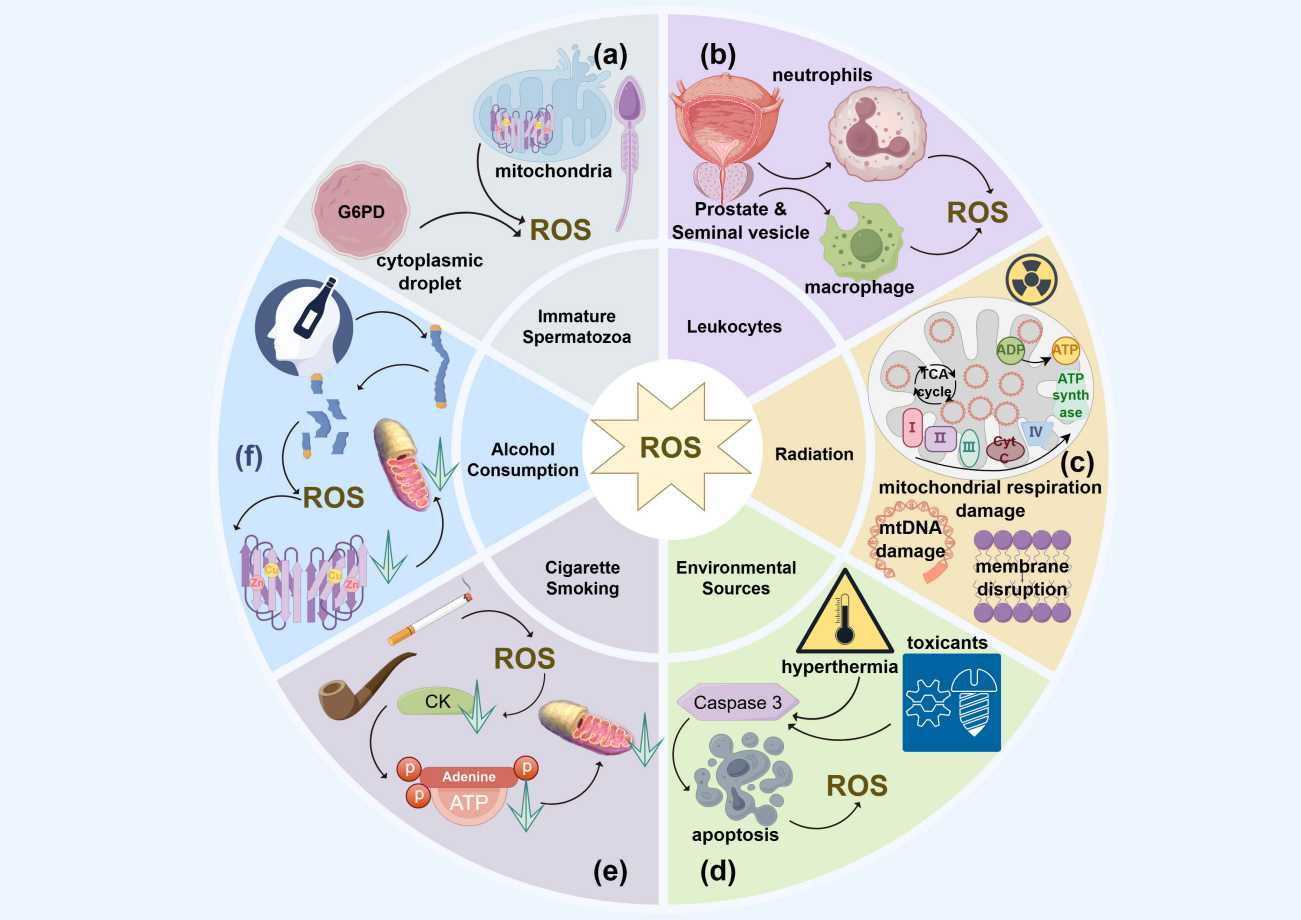
The endogenous and exogenous sources of ROS in idiopathic asthenozoospermia. **(a)** Immature spermatozoa; **(b)** Leukemia; **(c)** Radiation; **(d)** Environment resources; **(e)** Smoking; **(f)** Alcohol consumption.

A significant concentration of mitochondria is found in the midpiece of spermatozoa, serving as energy reservoirs that support sperm motility (108). The diaphorase enzyme, an oxidoreductase in the mitochondrial respiratory chain, maintains a balance between the oxidized and reduced forms of NADH to sustain sperm motility. However, a reduction in diaphorase enzyme activity can lead to superoxide generation, resulting in mitochondrial dysfunction through ROS-induced oxidative stress, potentially even damaging the mitochondrial integrity of human spermatozoa (109). Damage to the mitochondrial membrane by excessive ROS can further exacerbate ROS generation (110) (**Figure 2a**).

#### 4.1.2 Leukocyte

In patients with idiopathic asthenozoospermia, less than one million leukocytes per milliliter are typically found in naturally ejaculated semen (1). Most of the leukocytes from the prostate and seminal vesicles are activated leukocytes (106). Activated leukocytes, particularly peroxidase-positive types such as polymorphonuclear neutrophils (PMNs) and macrophages, are significant producers of ROS in human semen (111). These leukocytes enhance the production of NADPH, thereby increasing the activity of NADPH oxidases and resulting in elevated levels of superoxide O_2_^-^. Additionally, myeloperoxidase-positive neutrophils contribute to the oxidative conversion of O_2_ (112).

In addition, leukocyte-mediated signaling can also lead to an imbalance between oxidative and antioxidative processes. Elevated proinflammatory cytokines, such as interleukin (IL)-8, and reduced levels of superoxide dismutase (SOD) promote ROS generation, triggering oxidative stress. This stress, exacerbated by excessive leukocytes, ultimately damages spermatozoa (**Figure 2b**) (74).

### 4.2 Exogenous sources of ROS and their effects on sperm motility

The influence of environmental factors on sperm quality and motility represents a complex and multifaceted challenge. Over recent years, an expanding corpus of research has been dedicated to elucidating the mechanisms by which these factors impact male fertility, with particular emphasis on sperm quality and motility. This review specifically examines the roles of environmental factors, encompassing high temperatures, toxicants, and occupational exposures, as well as lifestyle factors, including obesity, smoking, alcohol consumption, and daily electronic radiation, in the generation of ROS and their impact on sperm motility.

#### 4.2.1 Environmental sources

Prolonged exposure to high temperatures and heat radiation can induce scrotal hyperthermia, promoting the generation of ROS. Studies have shown that high summer temperatures are associated with decreased sperm concentration and count, while variations in sunlight duration and humidity can also affect sperm quality (113). Research conducted in Argentina found that changes in sunlight duration and humidity are linked to reductions in sperm concentration, count, motility, and membrane integrity (113). The underlying mechanism involves the upregulation of Caspase 3, which induces apoptosis in Leydig and Sertoli cells of the human testis due to excessive ROS generated by heat stress (114, 115) (**Figure 2c**).

Chemical toxicants such as phthalates, originating from microplastic pollution, can lead to an overproduction of ROS in human spermatozoa and testicular germlines cells (116, 117). This condition is characterized by a reduction in testicular antioxidants and hormone levels, causing mitochondrial dysfunction and decreased sperm motility as a result of oxidative stress (116, 117). Similarly, heavy metal ions like cadmium, copper, iron, and lead can reduce sperm motility and affect other sperm parameters. These effects are attributed to mitochondrial DNA damage caused by excessive ROS (118–120). A study involving coke oven workers identified a dose-response relationship between exposure to metal mixtures and diminished sperm quality (121). Furthermore, air pollution can also impact sperm motility by compromising the integrity of the spermatic plasma membrane through excessive ROS production. Research conducted in southern China has revealed a significant association between exposure to air pollutants like CO, NO2, O3, PM10, and PM2.5 and reductions in sperm count and motility, particularly during critical periods of sperm development (122) (**Figure 2d**).

Occupational exposure and pesticides pose a global concern regarding male reproductive health, particularly in industrialized nations (12, 123). Research has indicated that exposure to environmental toxicants such as cadmium, mercury and bisphenol A (BPA) can lead to male infertility, a condition associated with oxidative stress (123). These toxicants instigate oxidative stress, thereby disrupting the normal function of reproductive cells and consequently affecting the quality and motility of sperm (124). For instance, cadmium and mercury can interfere with the intracellular antioxidant defense systems, leading to an overproduction of ROS, which in turn damage sperm DNA and membrane lipids (125). BPA and pesticides may mimic or disrupt endocrine functions, thereby affecting the balance of reproductive hormones and subsequently influencing spermatogenesis (126, 127). Therefore, reducing occupational exposure and the use of pesticides is crucial for the protection of male reproductive health.

#### 4.2.2 Lifestyle factors

Lifestyle factors and occupational exposures are considered significant influences on sperm quality. Studies indicate that obesity and irregular sleep patterns are associated with declines in sperm quality (128). In a study involving 1,060 participants, these lifestyle factors were significantly correlated with lower sperm quality (128). Research indicates that obesity leads to an increased accumulation of body fat, thereby triggering oxidative stress. This condition has a negative impact on sperm quality, particularly contributing to the occurrence of AZS (129). The oxidative stress induced by obesity not only affects the quality of sperm but may also exacerbate reproductive dysfunction by influencing the function of the reproductive axis (130). For instance, the disruption of tightly regulated metabolic pathways can lead to adverse reproductive outcomes, such as an inefficient energy supply to germ cells, defects in sperm motility, or arrest of spermatogenesis (129). Moreover, testicular metabolic alterations induced by obesity may also result in mitochondrial dysfunction, which is closely associated with the overproduction of ROS and oxidative stress readily targeting spermatozoa DNA and lipids, thereby contributing to a decrease in sperm quality (129).

Cigarette smoking is a major etiological factor in idiopathic asthenozoospermia. A meta-analysis of 20 studies conducted by Sharma et al. highlighted that cigarette smoking has an overall negative effect on sperm motility and other semen parameters (131). The accumulation of excessive ROS due to hazardous chemicals, carcinogens, and mutagenic substances in tobacco can damage mitochondrial DNA, inducing oxidative stress. Substances such as nicotine in cigarettes ultimately impair sperm motility by causing oxidative damage to the integrity of plasma membranes, altering protein and enzyme conformations and activation, and compromising the mitochondrial DNA sequence integrity. Creatine kinase (CK), a protein serving as a cellular energy reserve for fast ATP buffering and rebuilding in human spermatozoa, exhibits decreased activity in smokers. Elevated levels of reactive oxygen species (ROS) lead to additional oxidative damage to mitochondrial DNA, reducing ATP production and available energy, which, coupled with reduced CK activity, results in a rapid decline in sperm motility. Furthermore, the decline in sperm motility due to smoking is associated with protein phosphorylation, inhibition of histone-to-protamine transition, and disruptions in the expression of microribonucleic acids (miRNAs). Additionally, second-hand smoke also damages sperm motility through mitochondrial DNA damage and methylation, caused by excessive ROS (**Figure 2e**) (99, 100).

Sperm motility, concentration and morphology are deleteriously affected by excessive alcohol consumption as a result of spermatic chromatin abnormalities through apoptosis, oxidative stress for elevated ROS production and mitochondrial DNA damage (132). Ethanol from alcohol consumption leads to mitochondrial dysfunction and decreased ATP generation in hepatic metabolic processes (110). Cytochrome P450 enzymes (CYP2E), as a kind of catalyst for NADPH oxidase in oxidative stress, promote the concentrations of Cu^2+^ and Fe^3+^ under alcohol intake, which, through various pathways, enhance the generation of ROS (133). The generation of nitric oxide (NO) from inducible nitric oxide synthase (iNOS), which is secreted by macrophages, and its metabolite peroxynitrite will induce mitochondrial dysfunction under the stimulus of excessive ROS (**Figure 2f**) (134).

The biological impact of radiation on sperm motility is influenced by the type of radiation, as well as the dose and duration of exposure (135). In recent years, electronic devices such as mobile phones, computers, and microwave ovens have significantly increased exposure to ionizing radiation (136). It has been demonstrated that mobile phone radiation negatively affects the count, morphology, and motility of spermatogenic cells and spermatozoa (137). This radiation can damage the integrity of the plasma membrane and activate NADPH oxidase, leading to oxidative stress driven by elevated ROS and lipid peroxidation (LPO) (138). Electromagnetic radiation emitted by computers and mobile phones exerts adverse effects on sperm motility, capacitation, and acrosome reaction through oxidative stress induced by radiofrequency. This stress results from damage to mitochondrial DNA and disruptions in the electron transport chain within the mitochondrial respiratory complex (20). Additionally, sperm motility affected by oxidative stress is exacerbated by radiofrequency radiation due to decreased glutathione levels and compromised plasma membrane integrity (136). Furthermore, exposure to microwave radiation for two hours daily over 35 days has been shown to induce oxidative stress in human spermatozoa (**Figure 2c**) (139).

## 5 Therapeutic strategy of idiopathic athenozoospermia

Currently, there is no radical treatment for idiopathic asthenozoospermia that can fundamentally preserve sperm motility, primarily due to genetic alterations caused by ROS-mediated oxidative stress (140, 141). However, appropriate antioxidants and healthy lifestyle choices can help protect sperm motility (142). As previously mentioned, unhealthy lifestyle habits and endogenous sources such as immature spermatozoa and leukocytes contribute to excessive ROS in idiopathic asthenozoospermia (143–145).

**Figure 3:**
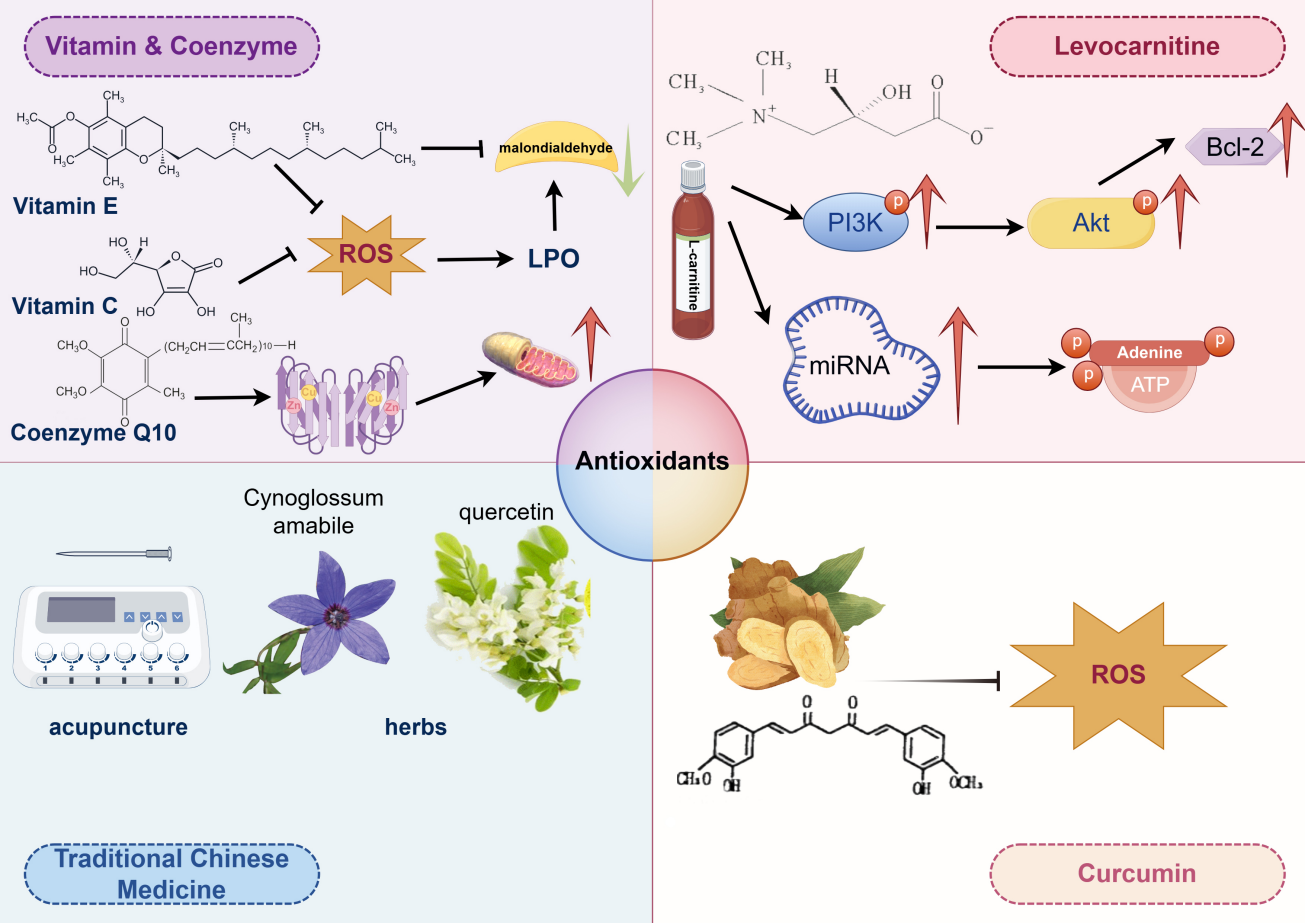
The therapeutic strategy of idiopathic athenozoospermia.

Antioxidants mainly function by suppressing ROS levels, inactivating ROS generated by metabolic processes and enzymatic reactions, thereby preventing lipid peroxidative damage to the plasma membrane of human spermatozoa (36, 37). Most antioxidants primarily have positive effects on reducing ROS levels (146), while a few can also repair oxidative stress damage in human spermatozoa caused by excessive ROS. Agarwal et al. found that approximately 85.6% of urologists and andrologists prescribed oral antioxidants to patients with abnormal semen parameters (147), demonstrating therapeutic effects on idiopathic asthenozoospermia (148, 149). Besides improving sperm motility, antioxidants may also upregulate the expression of fertility-associated sperm proteins in patients with idiopathic asthenozoospermia (150). Antioxidants protecting sperm motility include vitamins E and C, glutathione, hypotaurine, albumin, taurine, as well as superoxide dismutase (SOD) and catalase, while those elevating sperm motility are CoQ10 and N-acetyl cysteine (**Figure 3**) (12).

### 5.1 Vitamin C

Vitamin C, as an antioxidant, plays a critical role in alleviating oxidative stress, a recognized factor contributing to male infertility. It safeguards sperm from oxidative damage, thereby enhancing sperm quality and motility. Combination therapies that include Vitamin C have demonstrated promising results in improving sperm motility. For example, one study reported that while individual parameters such as sperm concentration and motility did not show significant changes, a regimen incorporating multiple antioxidants, including Vitamin C, significantly increased the total number of motile sperm (151). Another study emphasized the role of Vitamin C as an adjunct therapy following varicocelectomy, where it significantly improved sperm motility and morphology, highlighting its potential to enhance sperm quality post-surgery (152). Vitamin C effectively mitigates the adverse effects of environmental stressors, such as cigarette smoke and tetrahydrocannabinol exposure. It improved the motility and morphology of sperm exposed to cigarette smoke, underscoring its protective antioxidant properties (153). *In vitro* studies further demonstrated that Vitamin C could alleviate reductions in sperm motility and kinematics caused by tetrahydrocannabinol, further supporting its role in protecting sperm from various stressors (**Figure 3**) (153).

### 5.2 Vitamin E

Vitamin E serves as an oxygen radical scavenger, protecting sperm motility from reactive oxygen species (ROS)-mediated oxidative stress. It prevents the propagation of ROS, thus ensuring the integrity of the membrane and plasma lipoproteins of human spermatozoa (154). The level of malondialdehyde (MDA), a biomarker of lipid peroxidation (LPO) in oxidative stress, can be reduced by Vitamin E, thereby improving sperm motility (155). Additionally, Vitamin E can prevent DNA damage and fragmentation in human spermatozoa and their mitochondria caused by ROS (156, 157). Therefore, Vitamin E is potentially an effective treatment strategy for idiopathic asthenozoospermia, warranting a level B recommendation (**Figure 3**).

### 5.3 Coenzyme Q10

Coenzyme Q10 (CoQ10) primarily participates in the electron transport of oxidative phosphorylation during the respiratory process (158). It receives electrons from complex I and complex II, transferring them to complex III, to generate sufficient ATP necessary for maintaining sperm motility. Additionally, CoQ10 plays a role in transferring protons from fatty acids to the matrix (159). CoQ10 may positively influence nutrient uptake through the outer mitochondrial membrane, supporting the mitochondrial function of human spermatozoa (**Figure 3**) (160, 161).

### 5.4 Levocarnitine

Levocarnitine is a naturally occurring compound that has been demonstrated to enhance sperm motility, rendering it a promising candidate for the treatment of asthenozoospermia. The enhancement in sperm motility is attributed to several mechanisms, notably its role in energy metabolism and its influence on various molecular pathways. Specifically, levocarnitine upregulates the expression of PI3K, p-Akt, and BCL-2 proteins, thereby decreasing sperm cell apoptosis and improving both sperm count and motility (162). Additionally, levocarnitine modulates the expression of specific miRNAs, such as Hsa-mir-27b-3p and hsa-MIR-206, which are integral to energy metabolism pathways like ATP synthase activity and cAMP signaling. These pathways are crucial for sperm motility, providing a molecular foundation for the effectiveness of levocarnitine in the treatment of asthenozoospermia (163). In a randomized controlled trial, levocarnitine significantly enhanced sperm motility, morphology, and concentration when compared to coenzyme Q10 and vitamin E (164). It also increased testosterone and luteinizing hormone levels, suggesting a more extensive hormonal impact. A meta-analysis corroborated that levocarnitine and its derivatives substantially im-prove sperm motility and morphology relative to placebo, albeit without significant effects on serum hormone levels (165). Although levocarnitine demonstrates significant potential in treating asthenozoospermia, its precise molecular mechanisms remain partially elucidated, necessitating additional research to fully explore its capabilities and long-term effects. Moreover, while levocarnitine is efficacious, its combination with other therapeutic modalities may enhance its benefits, indicating that a multifaceted approach could be more beneficial for patients (**Figure 3**).

### 5.5 Curcumin

Curcumin, a natural compound derived from Curcuma longa (turmeric), exhibits numerous biological effects, including anti-inflammatory, antioxidant, anti-proliferative, and anti-metastatic activities. It is also recognized as a scavenger of reactive oxygen species (ROS) in both *in vitro* and *in vivo* settings (166, 167). Adequate levels of curcumin can enhance sperm motility by binding to promoters of antioxidant genes, thereby promoting the release of antioxidative enzymes and upregulating the expression of these genes to suppress ROS generation (158). It is also believed to aid in the cryopreservation of spermatozoa. However, excessive curcumin has been reported to mediate oxidative stress in the testes of rats. Notably, it is renowned as a potent non-steroidal contraceptive due to its ability to block sperm motility within the female reproductive tract (168). Therefore, the dosage of curcumin is crucial for regulating sperm motility (**Figure 3**).

### 5.6 Traditional Chinese medicine

The clinical application of Traditional Chinese Medicine in enhancing sperm motility involves a multifaceted approach combining herbal medicine, acupuncture, and integrative therapies. These methods have shown promising results in improving sperm motility and overall semen quality, providing a complementary treatment option for male infertility. One study indicated that acupuncture at the Fuxi point combined with tamoxifen citrate tablets significantly improved sperm motility parameters in patients with asthenozoospermia. This combination therapy enhanced sperm motility, average path velocity, and the percentage of motile sperm, outperforming tamoxifen alone (169). Cynoglossum amabile, a traditional Chinese herb, contains bioactive compounds with various pharmacological activities, including anti-inflammatory and cardiovascular effects. Although there is insufficient evidence for its direct application to sperm motility, its traditional use in treating reproductive issues suggests potential benefits (**Figure 3**) (170).

The multi-target approach of Traditional Chinese Medicine, involving compounds like kaempferol and quercetin, has been shown to regulate hormones, reduce oxidative stress, and improve sperm quality. These components are integral to the effectiveness of Traditional Chinese Medicine in treating male infertility (171). An integrated approach combining data mining, network pharmacology, and experimental validation has identified key components and mechanisms of Traditional Chinese Medicine prescriptions that enhance sperm motility. This approach underscores the multi-component, multi-target strategy of Traditional Chinese Medicine in treating male infertility (171).

While Traditional Chinese Medicine offers promising avenues for improving sperm motility, potential risks must be considered, such as hepatotoxicity associated with certain herbs like Cynoglossum amabile. Further research and clinical trials are needed to validate these therapies and ensure their safety and efficacy in broader applications (170).

## 6 The limitations and future prospects

Despite significant research into the relationship between ROS and sperm motility, several limitations have also persisted. For instance, accurately measuring ROS levels in semen and effectively assessing the efficacy of antioxidant treatments require further investigation (16). The short half-life of ROS poses challenges for their direct detection in human specimens (8). In addition, despite the availability of various antioxidant therapies for treating idiopathic asthenozoospermia, there is still no more effective clinical strategy to develop sperm motility. Furthermore, individual variability and the complex mechanisms of ROS and oxidative stress add to the challenges of research in this area (16). Consequently, future studies should delve deeper into the specific mechanisms by which oxidative stress affects sperm motility. The development of more effective diagnostic and therapeutic strategies is crucial to enhancing treatment outcomes for male infertility.

In summary, ROS plays a dual role in maintaining sperm motility. A moderate amount of ROS is essential for normal sperm function as they participate in energy acquisition, motility, and the capacitation process. However, excessive ROS can lead to oxidative stress, damaging the lipid bilayer structure of sperm membranes, impairing mitochondrial function, and affecting DNA integrity, which significantly reduces sperm motility and fertilization capacity. Therefore, maintaining an appropriate balance of ROS is crucial for ensuring male reproductive health. Further research should focus on exploring the potential benefits of antioxidant supplementation and its application in improving sperm quality and enhancing the effectiveness of male infertility treatments.
